# Golgi protein 73: charting new territories in diagnosing significant fibrosis in MASLD: a prospective cross-sectional study

**DOI:** 10.3389/fendo.2024.1506953

**Published:** 2025-01-13

**Authors:** Shan Hong, Ziyu Liu, Ping Li, Jing Zhang, Hongshan Wei

**Affiliations:** ^1^ Department of Gastroenterology, Beijing Ditan Hospital, Capital Medical University, Beijing, China; ^2^ Department of Gastroenterology, Beijing Xuanwu Hospital, Capital Medical University, Beijing, China; ^3^ Department of Liver Diseases, Beijing Youan Hospital, Capital Medical University, Beijing, China

**Keywords:** MASLD, metabolic diseases, liver biopsy, diagnosis, GP73

## Abstract

**Objectives:**

To explore the correlation between serum Golgi protein 73 (GP73) levels and the degree of fibrosis in Metabolic dysfunction associated steatotic liver disease (MASLD); to establish a non-invasive diagnostic algorithm based on serum GP73 and liver elasticity.

**Methods:**

This is a prospective cross-sectional study, including 228 patients diagnosed with MASLD from May 2018 to January 2024 at two tertiary hospitals. Clinical data and hepatic pathological features and the correlation between serum GP73 and liver fibrosis were assessed. A new algorithm was conducted after logistic regression. Receiver Operating Characteristic (ROC) curve was used to compare its diagnostic performance with traditional models.

**Results:**

Significant fibrosis was diagnosed in 37.2% (85/228) patients. Serum GP73 levels were markedly higher in patients with significant fibrosis than in those without (128 ng/mL v.s 46 ng/mL, p< 0.001). Serum GP73 levels independently predicted significant liver fibrosis (adjusted odds ratio, aOR 1.028, p< 0.001). A new algorithm based on GP73 was developed with a higher area under ROC (AUC) of 0.840 than that of Fibrosis index-4 (p< 0.001).

**Conclusions:**

Serum GP73 is an independent risk factor for significant liver fibrosis in MASLD, and the GFA (GP73-Fibroscan-Age) model has good diagnostic efficacy for significant liver fibrosis.

## Highlights

- Serum GP73 levels correlate with the severity of hepatic fibrosis in patients diagnosed with MASLD.
- Measurement of serum GP73 enhances the diagnostic accuracy for detecting significant fibrosis in individuals with MASLD.

## 1 Introduction

Metabolism-Associated Steatotic Liver Disease (MASLD), previously recognized as non-alcoholic fatty liver disease (NAFLD), ranks as the foremost liver disease etiology globally (1). The escalating prevalence, notwithstanding the majority of patients not progressing to liver-related complications, is a concerning trend (2). In certain regions, MASLD emerges as the principal cause of cirrhosis and the secondary indication for liver transplantation (3). The prevalence of MASLD, in accordance with the most recent findings from a retrospective, cross-sectional study conducted in regional China using data from the health management database between 2017 and 2022, is 36.91% (4). Due to a lack of sufficient diagnostic tools and effective pharmacological treatments, many patients remain undiagnosed and untreated (5). As MASLD progresses, it can precipitate a cascade of hepatic complications, starting with inflammation (metabolic dysfunction associated steatohepatitis, MASH), advancing through stages of liver fibrosis, and potentially culminating in cirrhosis (6). Since 2023, studies have demonstrated a high degree of consistency between MASLD and NAFLD (7, 8). Given this overlap, pathological grading, and other relevant concepts from NAFLD can be applied to MASLD. This approach allows us to leverage the well-established frameworks and insights from NAFLD research to better understand and define the characteristics of MASLD.

Liver fibrosis serves as a pivotal prognostic indicator, offering predictions for the progression towards cirrhosis and hepatocellular carcinoma (HCC) (9–13). Although liver biopsy is not one of the diagnostic criteria for diagnosing MASLD, the elucidation of histopathological characteristics facilitates a precise evaluation of the condition. It remains the best standard for the accurate staging of liver fibrosis and inflammation in most chronic liver diseases (14). However, the routine use of liver biopsy is limited by the risk of complications, sampling error (15). Since a liver biopsy is still needed to establish the diagnosis, accurate assessment of fibrosis stage is labor-intensive and prone to error. Moreover, many patients are reluctant to get biopsies because of the hazards involved, which include excruciating pain and serious complications. Due to the rising incidence of MASLD and the previously mentioned limitations of liver biopsy, a number of non-invasive tests (NITs) for precise staging and risk assessment have been developed. NITs that were initially developed for staging fibrosis, are also increasingly used to determine liver-related prognosis (6). The use of NITs in risk stratification to identify patients who are more prone to experience severe liver events shows increasing promise. The constraints of a biopsy in terms of patient stratification would be overcome by using markers that are more trustworthy than a biopsy. Prognostic markers that function optimally have the potential to someday take the role of a biopsy, support clinical decision-making, and make it easier to enroll patients who will benefit from clinical trial participation. The European Association for the Study of the Liver (EASL), the European Association for the Study of Diabetes (EASD), and the European Association for the Study of Obesity (EASO) Clinical Practice Guidelines propose using basic non-invasive panels such the NAFLD Fibrosis Score (NFS) and Fibrosis Index-4 (FIB-4) as part of the diagnostic process to rule out advanced fibrosis (16). According to a systematic review (17), the performance of FIB-4, NFS in risk stratifying patients for liver-related morbidity and mortality is comparable to that of a liver biopsy. However, their usage is limited by a significant proportion of false positives and false negatives (18–21). It is acknowledged that in order to track the advancement or regression of MASLD, new NIT are required (22).

Golgi Protein 73 (GP73), identified on the Golgi apparatus and alternatively termed Golgi membrane protein 1 or Golgi phosphoprotein 2, owes its nomenclature to its molecular weight of approximately 73 kDa (23). Predominantly localized to biliary epithelial cells, the efficacy of GP73 as a serum biomarker for HCC diagnosis surpasses that of the conventional marker alpha-fetoprotein in both sensitivity and specificity (24–29). Further research has found that serum GP73 levels increase with the progression of chronic liver disease and decrease as liver pathology improves (30). Emerging evidence supports the utility of serum GP73 as a fibrosis biomarker in chronic liver diseases (31, 32). Previous studies by our team shed light on the potential role of serum GP73 in reflecting disease severity in chronic hepatitis B (CHB) (33, 34). Currently, there are some single-center studies exploring the diagnostic accuracy of serum GP73 in MASLD patients (35). The diagnostic value of GP73 for MASLD merits further investigation.

In this context, this prospective study aims to: 1) quantify serum GP73 levels in biopsy-proven MASLD and evaluate their correlation with significant fibrosis;2) establish and validate a novel algorithm combining serum GP73 and TE for diagnosis of significant fibrosis in MASLD.

## 2 Subjects and methods

### 2.1 Study design

**Figure 1:**
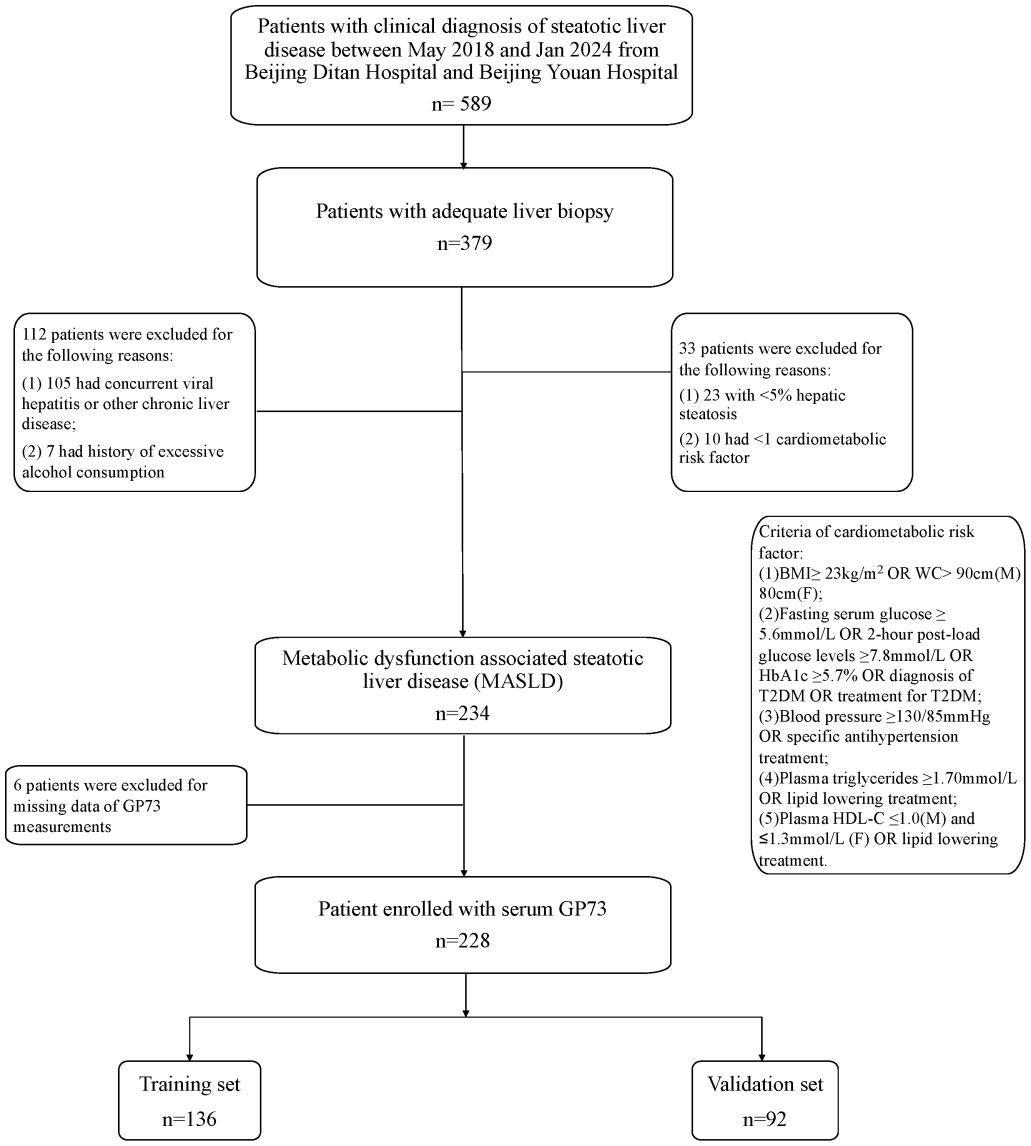
Study design. BMI, body mass index; T2DM, Type 2 diabetes; HbA1c, haemoglobin A1c; HDL-C, high-density lipoprotein cholesterol; MASLD, metabolic dysfunction associated steatotic liver disease; GP73, Golgi protein 73.

This dual-center, cross-sectional study prospectively recruited patients with diagnosis of steatotic liver disease in Beijing Ditan Hospital and Beijing Youan Hospital, Capital Medical University between May 2018 and Jan 2024 (**Figure 1**). Inclusion criteria were as follows: 1) the ability to give informed consent; 2) aged 18-75 years; 3) presence of hepatic steatosis detected by ultrasonography, computed tomography or other imaging techniques, and confirmed by liver biopsy. 4) patients with at least one of five cardiometabolic risk factors (CMRF). CMRFs were defined as follows: (1) Body mass index (BMI) ≥ 24kg/m^2^ OR Waist Circumference (WC) > 90 cm (M)/80 cm (F) (36, 37); (2) A fasting serum glucose ≥ 5.6 mmol/L OR 2-hour post-load glucose levels ≥ 7.8mmol/L OR haemoglobin A1c (HbA1c) ≥ 5.7% OR diagnosis of Type 2 diabetes (DM) OR treatment for DM; (3) Blood pressure ≥130/85 mmHg OR acceptance of any antihypertension treatment; (4) Triglycerides ≥ 1.70mmol/L OR specific lipid-lowering treatment; (5) Plasma High-Density Lipoprotein Cholesterol (HDL-C) ≤1.0 mmol/L for men and ≤1.3 mmol/L for women OR use of lipid-lowering treatment.

Exclusion criteria were as follows: 1) history of excessive alcohol consumption >30 g/day for men and >20 g/day for women) or other steatogenic drugs; 2) individuals of known other liver diseases (e.g., viral hepatitis, drug-induced liver disease or autoimmune liver disease); 3) individuals of known or suspicious malignant disease or HIV infection.

### 2.2 Ethics

The study was approved by the Ethics Committee of Beijing Ditan Hospital and Beijing Youan Hospital, and each participant provided written informed consent for the use of their data. All methods were performed following the relevant Declaration of Helsinki. This study has been registered with the China Clinical Trials Registry, under the registration number ChiCTR1800015157, to ensure the transparency and traceability of the research.

### 2.3 Demographic variables and laboratory parameters

Data acquisition, encompassing a wide range of parameters such as height, weight, waist circumference, blood pressure, medication history, and past medical conditions, was carried out close to the time of liver biopsy procedures, either on the same day or within 48 hours prior. The calculation of BMI for each participant was based on their height and weight measurements. Additionally, participants’ fresh samples underwent an extensive battery of standardized laboratory tests aimed at evaluating liver health and potential risk factors for associated diseases. This comprehensive panel included assessments for viral hepatitis, autoimmune antibodies, a full spectrum of liver function tests, complete blood count, coagulation function, lipid profiles, uric acid levels, blood glucose, and glycated hemoglobin. Liver stiffness evaluation was performed using Transient Elastography (TE, FibroScan) by an operator without knowledge of the participants’ clinical backgrounds. To ensure precise measurements across diverse body types, the examination utilized an M probe as standard, with an XL probe for individuals with a BMI ≥30 kg/m^2^. The procedure involved scanning the right liver lobe in a supine position with the right arm fully extended, aiming to achieve at least 10 valid readings. The median value of these readings was considered the final result, contingent upon the interquartile range to median ratio being ≤30%.

### 2.4 Quantitative detection of serum GP73

By applying a double-antibody sandwich enzyme-linked immunosorbent test (ELISA) kit (Hotgen Biotech Inc., Beijing, China), serum GP73 was quantitatively evaluated per the manufacturer’s instructions. To sum up, wells precoated with monoclonal anti-GP73 were filled with sample dilution buffer (50 μL) and 20 μL serum, then incubated for one hour at 37°C. After washing, the wells were incubated for 30 minutes at 37°C with 100 μL of horseradish peroxidase (HRP) -conjugated anti-human antibody. Tetramethylbenzidine was used to develop the plate after five washings, and a microplate reader (Spectramax M2; Molecular Devices, Sunnyvale, CA, USA) was used to measure the OD450. As a calibration reference, parallel assays were performed on purified recombinant GP73. Every assay was conducted twice in triplicate. In this study, all samples were subjected to two rounds of testing, conducted in August 2022 and January 2024, respectively. Each sample was tested twice to ensure the reproducibility and reliability of the results. The coefficient of variation during the testing process was maintained at less than 15% to ensure the precision of the experiment. All testing procedures were carried out by the same experienced technician to minimize the impact of operational variability on the outcomes, while also ensuring that the clinical information of the participants was kept confidential from the technician. According to the data provided by the reagent kit manufacturer, the upper reference limit for serum GP73 in a healthy population is 53 ng/mL.

### 2.5 Liver biopsy and histological evaluation

Percutaneous liver biopsies were performed with real-time transabdominal ultrasonography guidance. Masson trichrome and hematoxylin-eosin stains were applied to each specimen. A biopsy specimen was deemed adequate if it measured at least 10 mm in length and had six or more portal tracts. Three seasoned histopathologists who were blind to the clinical data reevaluated the presence of ballooning and NAFLD activity score (NAS) (38). The NAS is based on a standard grading system: steatosis (on a scale of 0-3), lobular inflammation (on a scale of 0-3), and ballooning (on a scale of 0-2). Finally, an agreement had to be set in. Patients were regarded as “missing data” if the opinions of the three pathologists’ evaluations were not in agreement. NAS ≥5 was used to define MASH. For analysis purposes, fibrosis stages 1a, 1b, and 1c were regarded as stage 1. Fibrosis stages 2-4 and 3–4 were used to define significant and advanced fibrosis, respectively.

### 2.6 Statistical analysis

Student’s t-test was used to analyze normally distributed data expressed as mean ± SD. The Mann–Whitney U or Kruskal–Wallis H test was used to analyze non-normal data which were shown as median (interquartile range). The chi-square test was used to analyze counts of categorical data. Spearman’s correlation was used to examine continuous variable relationships. A binary logistic regression analysis was conducted based on the presence of significant liver fibrosis as indicated by liver biopsy pathology. The sample was divided into a training group (60%, n=136) and a validation group (40%, n=92) using a random number generation method. Variables with a P-value of<0.10 in the univariate analysis were selected and incorporated into the multivariate model using a forward stepwise method. In the training group, we initially conducted univariate logistic regression analysis to select variables. Subsequently, we utilized multivariate logistic regression to further screen and refine the variables. Based on the results of the multivariate logistic regression, we formulated an equation, which constitutes our non-invasive diagnostic model. Based on the results of the multivariate logistic regression, which provided adjusted odds ratios and p-values for each variable, we formulated an equation. This equation constitutes our non-invasive diagnostic model, which was designed to predict the likelihood of significant liver fibrosis based on serum GP73 levels and other selected variables. This model was then evaluated for its diagnostic efficacy in the validation group. The diagnostic threshold (cut-off value) was determined by the maximum Youden index. The performance characteristics of the non-invasive diagnostic algorithm, including sensitivity, specificity, positive predictive value (PPV), and negative predictive value (NPV), were assessed. The diagnostic performance of the non-invasive diagnostic algorithms was evaluated by analyzing the area under the receiver operating characteristic (ROC) curves. The area under the curve (AUC) provides a measure of the overall performance of a diagnostic test, with values closer to 1 indicating higher diagnostic accuracy. Comparisons between the AUCs of different diagnostic algorithms were conducted to assess their relative performance in diagnosing the condition of interest. To compare the AUCs derived from the ROC curves, the DeLong et al. (39) method was employed, which provides a non-parametric approach to test for differences between two correlated ROC curves. This method is suitable for comparing the diagnostic accuracy of different tests applied to the same set of samples. A p-value of less than 0.05 was considered statistically significant for all analyses. Confidence intervals (95% CI) for the AUC were calculated to provide an estimate of the precision of the diagnostic accuracy measures. Additionally, sensitivity and specificity values were derived from the ROC curves to further describe the diagnostic performance of the algorithms at optimal cut-off points determined by the Youden index. A two-tailed P-value of<0.05 was considered statistically significant.

Sample Size Calculation: This is a single-sample diagnostic trial aimed at minimizing invasive procedures for patients. With an anticipated specificity of 80%, an allowable error of 0.1, a two-sided test, α=0.05, and a power of 1-β=0.9, the formula for calculating the sample size of a diagnostic study is as follows: Data were regarded as statistically significant when P< 0.05.


$$n={\left(\frac{Z_{1-\alpha /2}\times \sqrt{p_{0}\times \left(1-p_{0}\right)}+Z_{1-\beta }\times \sqrt{p\times \left(1-p\right)}}{\delta }\right)}^{2}$$

Z(1-α/2) and Z(1-β) are respectively represented by 1.96 and 1.28, as found in standard statistical tables. *p*_0_ denotes the anticipated specificity, while pindicates the minimum acceptable specificity. With the expected specificity (*p*) set at 0.8 and the acceptable lowest specificity (*p*_0_​) at 0.7, and accounting for a 10% loss to follow up, the study required the inclusion of at least 207 participants.

Statistical analyses were conducted using SPSS 25.0 (IBM Corp., Armonk, NY, USA), MedCalc Statistical Software version 19.2 (MedCalc Software Ltd, Ostend, Belgium) and GraphPad Prism 9.3.1 (GraphPad Software, San Diego, CA, USA).

## 3 Results

### 3.1 Study population

Between May 2018 and January 2024, the investigation was carried out at Beijing Ditan Hospital, Capital Medical University and Beijing You’an Hospital, Capital Medical University. During this period, 379 participants provided informed consent and underwent liver biopsy. Initial assessments revealed that 91.3% (346 individuals) fulfilled the criteria for MASLD. The study then excluded individuals with other conditions: 101 were diagnosed with chronic hepatitis B, 3 presented with drug-induced liver injury, 1 was identified with autoimmune hepatitis, and 7 exceeded the recommended alcohol intake levels. A further 6 individuals were removed from the cohort due to inadequate serum samples for GP73 analysis. Consequently, the study focused on a cohort of 228 MASLD patients as shown in **Figure 1**.

### 3.2 Clinical and pathological characteristics

Among the patients meeting MASLD criteria, men represented 57.5% (131/228) as shown in **Table 1**. Significant fibrosis was diagnosed in 37.2% (85/228) patients. Higher BMI or waist circumference emerged as the predominant CMRF. Specifically, 54 participants (23.7%) exhibited at least one CMRF, whereas 103 participants (45.7%) had three or more CMRFs.

**Table 1:** Comparison of clinical characteristics of patients with metabolic associated fatty liver disease by fibrosis staging.

Characteristic	No Significant Fibrosis (n=143)	Significant Fibrosis (n=85)	p-value	Total (n=228)
Age (years)	38 (30-48)	45 (31-57)	0.025	40 (30-52)
Female, n (%)	55 (38.5%)	42 (49.4%)	0.017	97 (42.5%)
ALT (U/L)	72 (42-164)	92 (50-206)	0.216	83 (48-175)
AST (U/L)	54 (33-128)	81 (42-149)	0.044	63 (35-143)
PLT (10^9/L)	209 (164-259)	199 (154-251)	0.305	206 (165-256)
Total Cholesterol (mmol/L)	4.74 (4.09-5.41)	4.86 (4.34-5.60)	0.606	4.80 (4.22-5.55)
TG (mmol/L)	1.46 (0.98-2.17)	1.74 (1.20-2.73)	0.625	1.56 (1.05-2.45)
HDL-C (mmol/L)	1.06 (0.90-1.21)	1.02 (0.84-1.32)	0.768	1.04 (0.92-1.22)
LDL-C (mmol/L)	3.04 (2.66-3.92)	3.23 (2.53-3.90)	0.333	3.07 (2.61-3.90)
LSM (kPa)	6.1 (5.3-7.1)	7.6 (4.6-12.8)	<0.001	7.0 (5.5-9.2)
GP73 (ng/mL)	46.1 (32.8-77.1)	128.2 (80.9-183.6)	<0.001	66.1 (38.5-130.8)

ALT, Alanine Aminotransferase; AST, Aspartate Aminotransferase; HDL-C, High-Density Lipoprotein Cholesterol; LDL-C, Low-Density Lipoprotein Cholesterol; LSM, Liver Stiffness measurement; PLT, Platelet Count; GP73, Golgi Protein 73; TG, Triglycerides

Values are presented as median (interquartile range) or number (percentage) as appropriate.

### 3.3 Association between significant fibrosis and serum GP73 levels

**Figure 2:**
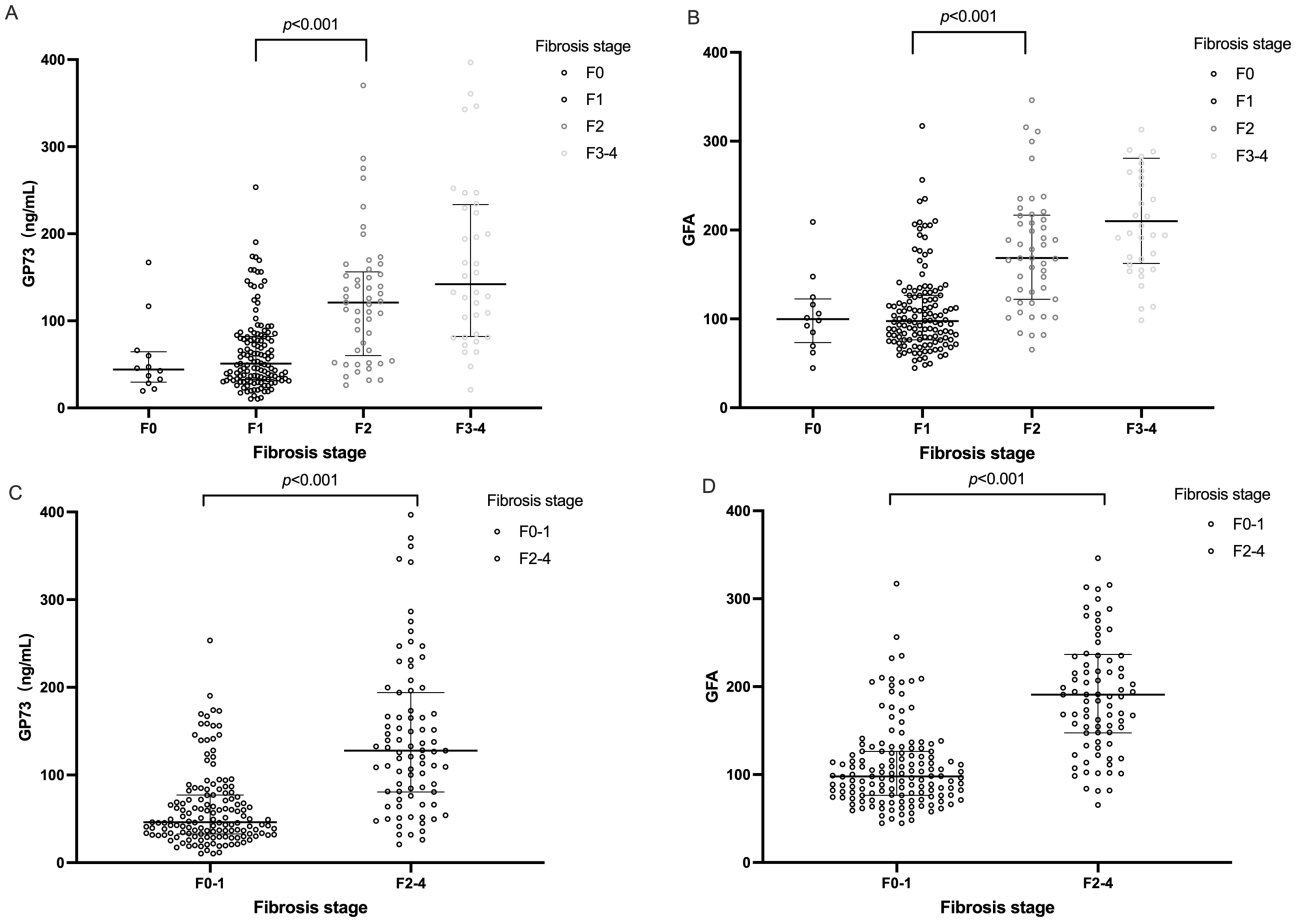
Distributions of serum GP73 value and GFA score according to fibrosis stage. The horizontal bar inside the scatterplots represents the median value. Comparisons between each two groups were performed using Mann–Whitney U test. **(A)** Distributions of serum GP73 levels according to fibrosis stage. **(B) **GFA score values according to fibrosis stage. **(C)** Distribution of serum GP73 levels in patients with and without significant hepatic fibrosis. **(D)** Distribution of GFA score values in patients with and without significant hepatic fibrosis. Due to the small sample size of the F4 fibrosis stage, we combined and presented the samples from stages F3-F4 for analysis. GFA= -8.757+1.048\times Agey+1.028×GP73ng/mL+1.588×LSM(kpa).

The study participants were categorized based on the degree of liver fibrosis into two groups: those with significant fibrosis (F≥2) and those without significant fibrosis (F0-1) for comparative analysis. The findings indicated that patients with significant fibrosis were older on average (45 years v.s 38 years, p= 0.025) and had a higher proportion of females (49.4% v.s 38.5%, p= 0.017). With the progression of liver fibrosis, GP73 levels gradually increase. Additionally, serum GP73 levels were markedly higher in patients with significant fibrosis than in those without (128 ng/mL v.s 46 ng/mL, p< 0.001) as shown in **Figure 2**. However, no statistically significant differences were observed between the two groups in terms of ALT or HDL-C. A correlation coefficient of 0.269 (p< 0.001) was identified between GP73 levels and NAS.

### 3.4 Pathological characteristics

Liver fibrosis was graded as F0-1 in 143 cases, accounting for 62.7%; F2 was observed in 49 cases, representing 21.5%; and patients reaching advanced liver fibrosis (F3-4) totaled 36, comprising 15.8% of the cohort. Consequently, the proportion of patients diagnosed with significant liver fibrosis (F2-4) was 37.3%. A diagnosis of MASH was made in 80 cases (35.1%). A statistically significant correlation was identified between the diagnosis of MASH and the presence of significant liver fibrosis, with a Pearson correlation coefficient of 0.402 (p< 0.001). The AUC for MASH in predicting significant liver fibrosis was 0.699 (95% CI: 0.626-0.772).

Furthermore, patients were stratified into three groups based on the number of CMRFs they possessed (1, 2, ≥3) to investigate the relationship between the quantity of risk factors and the incidence of significant liver fibrosis. The incidence of MASH significantly escalated with an increasing number of risk factors (**Supplementary Figure S1**), recorded at 20.4%, 23.9%, and 50.5%, respectively, with this difference being statistically significant (p< 0.001).

### 3.5 GP73 as an independent predictor of significant liver fibrosis

The degree of liver fibrosis (stages F2-4) was served as the positive outcome of interest in training set (n=136). Univariate and multivariate logistic regression analyses were conducted, and factors associated with significant liver fibrosis were identified. The analysis indicated that patient age, liver stiffness measurement (LSM) values, and serum GP73 levels each independently predicted significant liver fibrosis as shown in **Table 2**. Based on the results of the multivariate analysis, we developed a novel non-invasive diagnostic algorithm, named “GFA” (GP73-Fibroscan-Age), which integrates information on GP73, LSM, and age. The formula for the GFA algorithm is as follows:

**Table 2:** Univariate and multivariate logistic regression analysis results for significant liver fibrosis.

Variable	Significant fibrosis			
	Univariate analysis		Multivariate analysis	
	OR (95%CI)	*p-*value	aOR (95%CI)	*p-*value
Male gender		0.096		
Age		0.072	1.048 (1.005-1.092)	0.029
BMI		0.053		
ALT		0.390		
AST		0.380		
ALB	0.874 (0.811-0.943)	<0.001		
TG		0.988		
HDL-C		0.059		
GP73	1.020 (1.012-1.028)	<0.001	1.028 (1.017-1.039)	<0.001
PLT		0.374		
LSM	1.496 (1.254-1.784)	<0.001	1.588 (1.306-1.931)	<0.001

BMI, Body Mass Index; ALT, Alanine Aminotransferase; AST, Aspartate Aminotransferase; HDL-C, High-Density Lipoprotein Cholesterol; LSM, Liver Stiffness measurement; PLT, Platelet Count; GP73, Golgi Protein 73; TG, Triglycerides; ALB, albumin


−8.757+1.048×Agey+1.028×GP73ng/mL+1.588×LSM(kpa)

Consistent with GP73 levels, the GFA algorithm values show a gradual increasing trend with the progression of liver fibrosis stages as shown in **Figure 2**.

### 3.6 Comparison of diagnostic accuracy between the GFA model and traditional NITs

**Figure 3:**
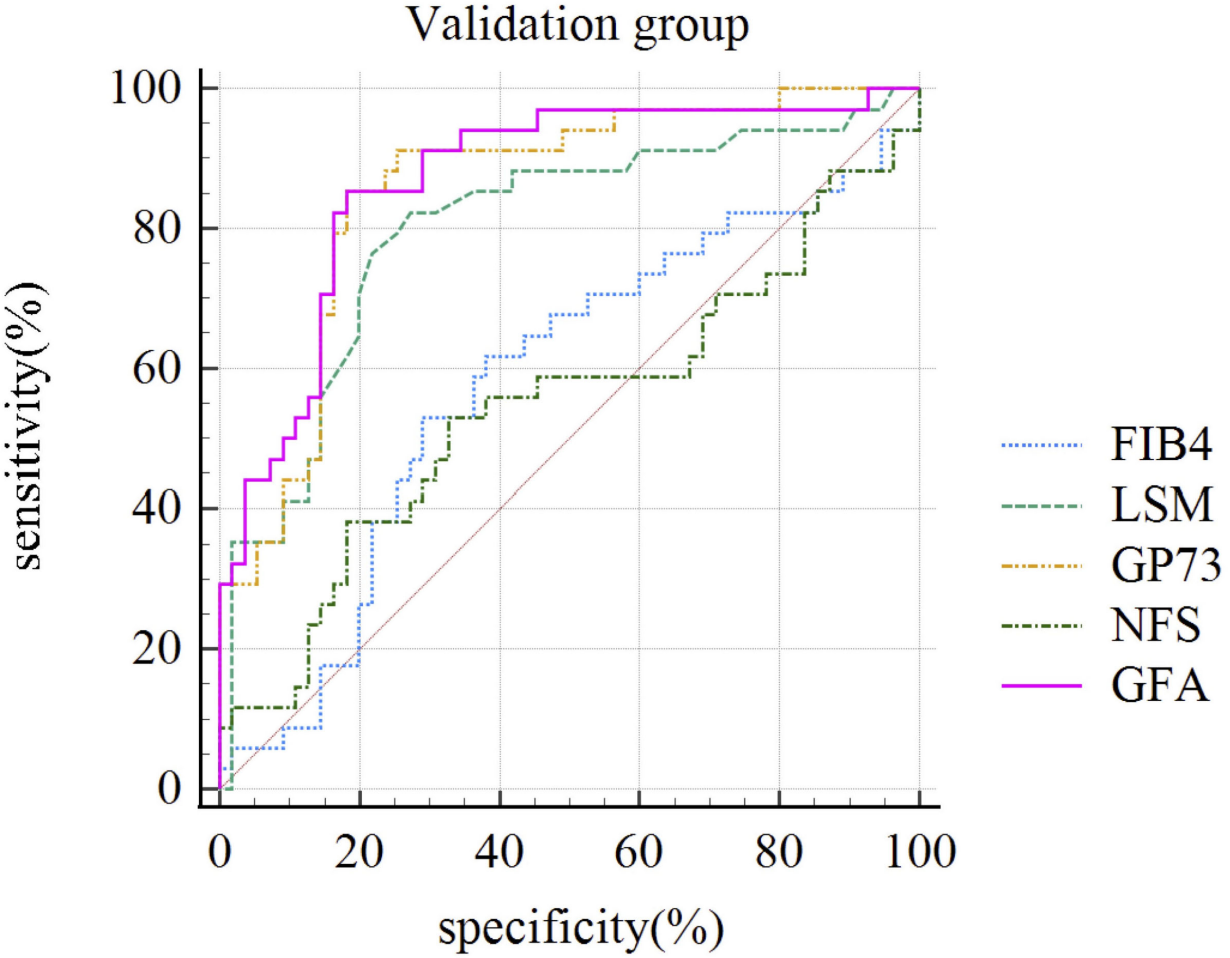
ROC Curves of Different Non-Invasive Diagnostic Models for Diagnosing Significant Liver Fibrosis in the Validation Group (n=92). FIB-4, Fibrosis index-4; LSM, liver stiffness measurement; GP73, Golgi protein 73; NFS, NAFLD Fibrosis Score; GFA, GP73-Fibroscan-Age.

To further compare the diagnostic performance of the new diagnostic algorithm with traditional algorithms, we compared the diagnostic performance of GFA, which was based on LSM and GP73, and serological-based models such as FIB-4 and NFS levels across the entire sample by constructing ROC curves. The AUC for the GFA model was 0.860 (95% Confidence Interval: 0.811-0.909). The difference in AUC between the GFA model and liver stiffness measurements (an incremental increase of 0.070) did not reach statistical significance (p=0.301). However, the diagnostic performance of the GFA model was significantly superior to that of the FIB-4 and NFS (p< 0.001). This conclusion was also reflected in the comparison within the validation group (92/228), where the GFA model’s AUC for significant liver fibrosis was superior to LSM, FIB-4, and NFS as shown in **Table 3** and **Figure 3**.

**Table 3:** Comparison of performance of different non-invasive diagnostic models in diagnosing significant liver fibrosis in validation set.

Model	AUC (95% CI)	Sensitivity (%)	Specificity (%)	PPV (%)	NPV (%)	P-value (compared to GFA)
LSM	0.807 (0.711-0.881)	82.86	73.68	65.90	87.50	0.286
GFA	0.840 (0.748-0.908)	82.86	82.46	74.36	88.68	–
NFS	0.527 (0.419-0.632)	52.94	64.91	48.08	69.20	<0.001
FIB-4	0.575 (0.466-0.679)	51.43	70.91	52.94	69.64	<0.001

AUC, Area Under the Receiver Operating Characteristic Curve; CI, Confidence Interval; LSM, liver stiffness measurement; NFS, NAFLD Fibrosis Score; FIB-4, Fibrosis-4; GFA, GP73-Fibroscan-Age; PPV, Positive Predictive Value; NPV, Negative Predictive Value. Values are presented with sensitivity, specificity, positive predictive value, and negative predictive value percentages. P-values are calculated in comparison to the GFA model.

## 4 Discussion

This study prospectively established a non-invasive diagnostic algorithm based on GP73, and for the first time compared the diagnostic efficacy of the GP73-based diagnostic algorithm and conversional NITs for significant liver fibrosis in MASLD patients confirmed by liver biopsy. A key finding of the study is that GP73 is an independent risk factor for significant liver fibrosis in MASLD.

The first hallmark of our study is that serum GP73 was independently associated with higher risk of fibrosis. Zheng et al., 2020 (40) conducted serum GP73 and cytokeratin-18 M30 fragment testing on 105 patients with biopsy-confirmed MASLD despite persistently normal alanine aminotransferase levels, establishing a non-invasive diagnostic algorithm for nonalcoholic steatohepatitis (NASH) using both markers sequentially, achieving an accuracy of 82.9%. Current research on the correlation between GP73 levels and liver fibrosis in the MASLD population is scarce. A study with a sample size of 91 (35) found a positive correlation between serum GP73 concentration and significant liver fibrosis in MASLD, consistent with our findings. A systematic review analyzed the sensitivity and specificity of GP73 for liver fibrosis in chronic liver disease as 0.63 (95% CI = 0.60–0.65) and 0.79 (95% CI = 0.76–0.81), respectively, with an AUC of 0.818, although the study did not perform stratified analysis by different liver disease etiologies (41). In a retrospective analysis of 497 chronic hepatitis B virus-infected individuals (42), found a significant correlation between serum GP73 levels and liver fibrosis staging (r=0.539). A retrospective analysis of untreated chronic hepatitis B found a positive correlation between serum GP73 levels and liver fibrosis grading, unaffected by e antigen, HBV DNA viral load, or ALT levels (43). This study using 267 cases for model development and validated in a group of 133 confirmed that serum GP73’s diagnostic performance was superior to FIB-4 (AUC 0.76 vs. 0.66) and combining G73 with liver elasticity achieved an AUC of 0.85. These results above all suggest the significant diagnostic value of GP73 in liver fibrosis.

Elevated serum GP73 levels were found to have a good correlation with MASH in our study, and MASH has good consistency with the diagnosis of significant liver fibrosis (69.9%), with patients with severe liver inflammation showing elevated serum GP73. A point of debate centers on the source of elevated GP73 levels in MASLD patients: is it hepatocyte damage or liver fibrosis that primarily contributes to this increase? Iftikhar et al. (44) revealed that the main trigger for GP73 expression is progressive tissue remodeling and fibrogenesis in chronic liver disease. Many studies including our previous research have explored that serum GP73 concentration is correlated with liver fibrosis. While attempting to clarify the mechanisms underlying, efforts are also being made to disseminate and implement the findings in clinical settings (38, 45–48). Intracellularly, GP73 expression is influenced by transcriptional and post-translational modifications, including Furin-mediated cleavage. *In vitro*, serum GP73 levels possibly upregulated under inflammatory conditions (49) and *in vivo*, the elevation of serum GP73 level was triggered by liver inflammatory injury (50). Extracellularly, GP73 levels are influenced by factors such as inflammation, liver injury, and changes in liver function that affect protein shedding and clearance. Within the spectrum of chronic liver diseases, the expression levels of GP73 protein and mRNA rise progressively, manifesting in both hepatocytes and in stellate cells that have been activated, with the latter being particularly implicated in the development of liver fibrosis (51, 52). Whether its influence interferes with the diagnosis of liver fibrosis still requires further stratified analysis of inflammation levels, which affect the accuracy of this serum marker as a single biomarker for significant liver fibrosis.

The second hallmark our study is that a new algorithm was conducted with a good diagnostic accuracy. Currently, serological markers for liver fibrosis are divided into two types: indirect and direct. Indirect serum markers include combinations of routine laboratory parameters and demographic data such as age (53). Recently, Agile 3+ and 4 scores were established, combining LSM with inherent demographic data (age, sex, and the presence of type 2 diabetes) and serum biomarkers (ALT, AST, and platelets) to better diagnose advanced fibrosis and cirrhosis in MASLD, with diagnostic efficacy AUC of 0.85, similar to that of LSM (AUC 0.83, p= 0.142) (54–57). This algorithm is similar to the GFA explored in this study, and both are significantly superior to FIB-4, but its calculation method is more complex, and the inclusion of more reference indicators limits its application. GFA can be simply applied using a single serological and liver elasticity measurement, which is easy to operate. Serum GP73 can be measured using commercial ELISA kits, which can be established in hospitals or clinics with biochemical laboratories, with the cost of a single test only about 100 RMB and can be conducted simultaneously with other liver function tests. The GFA model only requires knowledge of the patient’s age, LSM, and GP73 level, low cost, and high accuracy in predicting significant liver fibrosis, and dynamic monitoring may also predict the occurrence of liver cancer, with further cost-effectiveness ratio and predictive effect on prognosis that can be quantified by subsequent research.

There is a growing recognition of the intricate interplay between metabolic risk factors such as obesity, diabetes, chronic kidney disease, and cardiovascular disease, highlighting their shared pathophysiological underpinnings (58). Though MASLD is prevalent as a chronic liver condition often progressing to cirrhosis, empirical evidence suggests that cardiovascular issues are the leading contributors to mortality in patients with this disease (59). Previous study provides significant evidence that GP73 is causally associated with incident coronary artery disease and other atherosclerotic events sharing similar etiology based on proteomics and 2-sample Mendelian randomization design, suggesting its role in the pathogenesis of coronary artery disease (60). Elevated vascular GP73 expression has been validated through the examination of both murine models and human tissue samples. In the realm of translational medicine, it is anticipated that forthcoming foundational studies that substantiate and elucidate the fundamental pathological mechanisms of GP73 or the GFA algorithm proposed in this study based on GP73 could contribute to the development of efficacious strategies for the management of MASLD.

The incorporation of these non-invasive techniques holds great potential to transform the diagnostic environment as the field develops, enabling prompt interventions and enhancing patient outcomes for MASLD patients. Patients with MASLD can now benefit from precise diagnosis and risk evaluations without invasive liver biopsies due to the development of reliable NIT based on GP73. Future studies will further explore whether GP73 could serve as a valuable prognostic tool for assessing the risk of liver-related events and overall mortality.

The limitations of the study include the following: This is a dual-center study, with research samples from two tertiary hospitals focusing on liver disease, and all patients underwent liver biopsy, as patients undergoing liver biopsy in large hospitals are usually considered to have a higher risk of adverse outcomes, which may have caused selection bias. To minimize potential variability related to differences in sample storage duration, an interim analysis was performed in August 2022, followed by a final assessment in January 2024, after the completion of sample collection. These measures were taken to mitigate any effects associated with prolonged serum storage, ensuring consistency in the data. Due to the current lack of widespread testing for GP73, the clinical sample size is limited, and our new algorithm has not been externally validated. However, despite the small sample size, the percentage of significant liver fibrosis and demographic characteristics are very similar to those of previous larger sample MASLD studies conducted by our team (7), suggesting that the results of this study can be extrapolated and generalized. Furthermore, due to the small number of cases of significant liver fibrosis (n=85), further stratified analysis of the population for the proportion of positive outcomes could not be conducted. Therefore, further large-scale, multi-center prospective validation of this diagnostic algorithm is needed before it can be widely applied.

## 5 Conclusion

In summary, serum GP73 is an independent risk factor for significant liver fibrosis in MASLD, and the GFA model based on GP73 combined with LSM has good diagnostic efficacy for significant liver fibrosis, effectively reducing the need for liver biopsy pathology in MASLD patients.

## Data Availability

The raw data supporting the conclusions of this article will be made available by the authors, without undue reservation.
