# Supplementary material for: Golgi protein 73: charting new territories in diagnosing significant fibrosis in MASLD: a prospective cross-sectional study
Source: Front Endocrinol (Lausanne). 2025 Jan 13;15:1506953. doi: 10.3389/fendo.2024.1506953 (PMC11769827; doi:10.3389/fendo.2024.1506953)
Supplement: Supplementary file 1 [file DataSheet1.docx]

**Supplementary material**

**
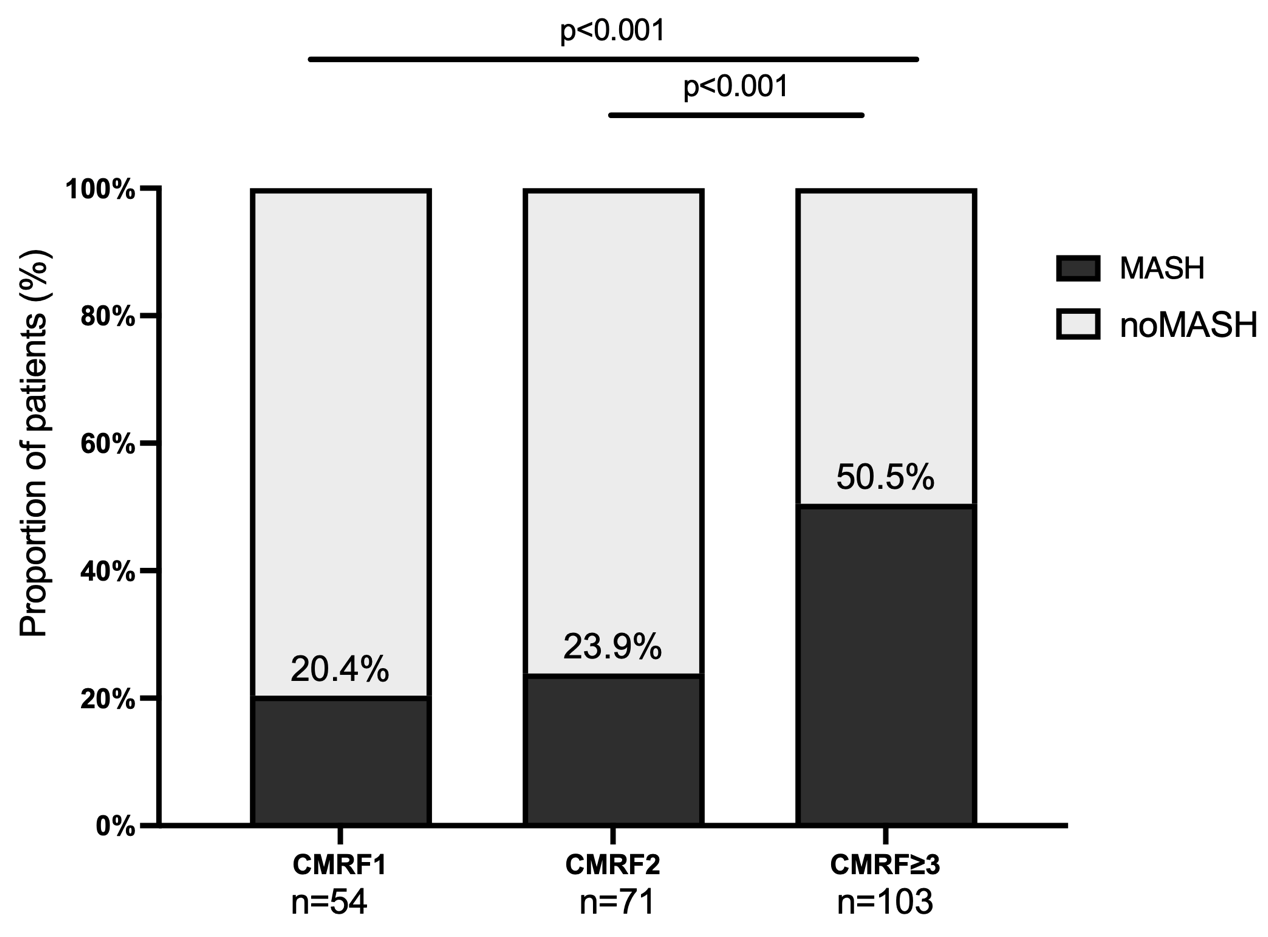
**

**Figure S1: Relationship Between the Number of Combined CMRF and the Incidence of MASH**

CMRF, cardiometabolic risk factor; MASH, metabolic associated steatohepatitis
